# Process for mainstreaming genetic cascade testing into primary and tertiary health systems in familial hypercholesterolaemia (FH), an autosomal dominant, fully penetrant disorder

**DOI:** 10.1136/fmch-2024-003258

**Published:** 2025-08-14

**Authors:** Karen L Birkenhead, David Sullivan, Madeline Calder, Catherine Spinks, Gabrielle Fleming, Claire Trumble, Cameron Hemmert, Ronald Trent, Shubha Srinivasan, Kerrie Martin, Bridie Carr, Charlotte M Hespe, Mitchell Sarkies

**Affiliations:** 1 School of Health Sciences, Faculty of Medicine and Health, The University of Sydney, Sydney, New South Wales, Australia; 2 Implementation Science Academy, Sydney Health Partners, Sydney, New South Wales, Australia; 3 Department of Chemical Pathology, Royal Prince Alfred Hospital, Sydney, New South Wales, Australia; 4 Central Clinical School, Faculty of Medicine and Health, The University of Sydney, Sydney, New South Wales, Australia; 5 Institute of Precision Medicine and Bioinformatics, Sydney Local Health District, Sydney, New South Wales, Australia; 6 Department of Medical Genomics, Royal Prince Alfred Hospital, Sydney, New South Wales, Australia; 7 Institute of Endocrinology and Diabetes, The Children’s Hospital at Westmead, Sydney, New South Wales, Australia; 8 Discipline of Child and Adolescent Health, Faculty of Medicine and Health, The University of Sydney, Sydney, New South Wales, Australia; 9 Clinical Genetics Network and Ophthalmology Network, Agency for Clinical Innovation, Sydney, New South Wales, Australia; 10 Cardiac Network, Agency for Clinical Innovation, Sydney, New South Wales, Australia; 11 School of Medicine, University of Notre Dame Australia, Sydney, New South Wales, Australia

**Keywords:** Physicians, Primary Care, General Practice, Genetics, Medical, Health Services Research, Cardiovascular Diseases

## Abstract

**Introduction:**

Advances in clinical genomics have raised the importance of integrating genomic medicine across healthcare systems, including primary care. Primary care presents an ideal environment to offer equitable and efficient access to genetic services. Familial hypercholesterolaemia (FH) is a preventable and treatable cause of premature heart disease and represents a health condition that can be successfully diagnosed and managed in primary care. This study describes a process for tailoring a primary-tertiary shared care model for FH to optimise health professional and patient engagement.

**Methods:**

Data were collected through semistructured interviews (n=10) with stakeholders in New South Wales, Australia. Interviews gathered feedback on how to tailor a shared care model for FH between tertiary and primary care services. Reflexive thematic analysis was used to analyse interview transcripts.

**Results:**

Analysis generated three main themes: (1) current process for genetic testing and management, (2) challenges with genetic testing for FH in primary care and (3) components needed to enable a tertiary-initiated shared care model. Participants considered the model of care acceptable and could be successfully implemented, provided key supports were in place to assist general practitioners. Based on these results, a process model for integrating genetic testing for other conditions into primary care settings was developed, using FH as an exemplar.

**Conclusion:**

The process model for tailoring of a primary-tertiary model of care for FH can be applied across a range of primary care services and treatable genetic conditions.

**WHAT IS ALREADY KNOWN ON THIS TOPIC**

- Primary care settings are an ideal environment to integrate genomic medicine into routine practice and help improve access to genetic services for all individuals.

**WHAT THIS STUDY ADDS**

- This study provides a process model for integrating genetic testing into primary care settings and demonstrates how primary and tertiary care providers can successfully work together to improve access to genetic services for common autosomal dominant genetic conditions such as familial hypercholesterolaemia.

**HOW THIS STUDY MIGHT AFFECT RESEARCH, PRACTICE OR POLICY**

- Future research should include implementation science approaches to help understand and address factors that may impact the integration of genomic medicine into routine practice for other conditions and help optimise patient outcomes, enhance equity in access to genomic services and ensure sustainability across healthcare systems.

## Introduction

 Substantial advances in genomic medicine have enabled more personalised care and improved patient outcomes.^1^ Advancements in technology, treatment approaches and direct-to-consumer genetic testing platforms have increased demand to integrate genomic medicine into existing health systems.^2^ Genomic medicine is rapidly expanding and, as such, exceeding the capacity for genetic specialists to meet demand.^3^ There is a need to mainstream the provision of genomic medicine to other medical specialties, including primary care.

General practitioners (GPs) are usually the first point of contact and a key stakeholder in the continuity of patient care, playing an integral role in the implementation of genomic medicine into health systems.^4^ Primary care presents an ideal setting to access genomic medicine in a more equitable and efficient manner, especially for those living in rural, regional or remote areas where access to specialist genetic services is limited.^5^ However, several barriers to integrating genomic medicine into primary care have been described, including limited knowledge of genetics, a lack of confidence in providing genetic care and shortage of resources and specialist support.^5^,^7^ GPs manage a range of health concerns daily that require knowledge across multiple health conditions, including genetic disorders. Previous research indicates that adequate support for GPs to integrate genomic medicine into practice includes contact information for local genetic services, genetic testing and referral information, educational resources and two-way communication with genetic specialists.^8^ Preparing GPs to deliver genetic services as part of routine care requires more than providing education and training and, instead, a collaborative, system-wide approach that engages multiple stakeholders is needed.^9^ Efficient, equitable access to genomic medicine through primary care is expected to benefit patients, families, populations and the health system.^10 11^

Familial hypercholesterolaemia (FH) is an autosomal dominant inherited disorder of lipid metabolism that leads to elevated low-density lipoprotein (LDL) cholesterol levels from birth. Left untreated, FH causes premature atherosclerotic cardiovascular disease (ASCVD) and death.^12^ Although a highly preventable cause of premature cardiovascular disease, FH remains poorly diagnosed and poorly treated, with less than 10% of the greater than 35 million people worldwide with the condition being diagnosed.^13^ Siblings, children and parents of someone diagnosed with FH have a 50% chance of also having the condition.^14^ FH is a Tier 1 genomic application, meaning it is a preventable cause of ASCVD and death, supported by strong evidence-based guidelines, and significant potential to have a positive impact on public health. Additionally, FH can be easily diagnosed and managed in primary care, provided appropriate supports are in place.^15^

As a common genetic condition, FH represents an ideal case study for integrating genetic testing into primary care, as a DNA genetic test is available that allows the commonly affected gene (*LDLR*), and a limited number of other genes, to be studied.^16^ Interpreting the genetic results is not always straightforward and will require additional support for GPs to be involved. Genetic counselling for FH does not require the level of complex decisions around treatment in comparison to other genetic conditions such as for BRCA1- and BRCA2-associated hereditary breast and ovarian cancer.^17^ Finally, the management of FH is well within the scope of practice for GPs (eg, cholesterol is commonly managed by GPs). An important goal of FH treatment is the prevention of early onset ASCVD, and as primary prevention is a fundamental component of primary care, GPs play an important role in the diagnosis, care and management of this common genetic condition.

The International Atherosclerosis Society guidelines for implementing best practice describe the optimal diagnosis and management of FH, which include the use of cascade genetic testing (testing of at-risk relatives of an individual with genetically confirmed FH).^18^ In Australia, Medicare Benefits Schedule (MBS) Items for genetic testing were introduced in 2020 which allow GPs to undertake cascade genetic testing. Additionally, the integrated guidelines for the care of FH in Australia were released and highlight the importance of models of care that can be adapted to local context.^12^ Since the release of these updated guidelines and the new MBS Items, there have been renewed efforts to implement improved detection of FH. Informed by implementation science methods, a primary-tertiary shared care model was developed for cascade genetic testing in primary care settings.^19^ Currently, there are limited models of care for integrating genomic medicine into primary practice that can be tailored across primary care settings. As such, practical models of care that cover the patient journey from diagnosis to management that can be tailored to specific settings are needed to help GPs effectively integrate genomic medicine into practice. This study describes a process model for the tailoring and implementation of a model of care for genetic testing in primary care settings in New South Wales, Australia. The purpose of the paper is to describe a step-by-step process of tailoring a shared care model for FH to local needs. The study aims were to (1) gather participant insights on how the model of care would work within local settings and identify elements or supports needed to ensure success of the model and (2) use the findings to develop a generic model of care for integrating genomic medicine for other common and treatable autosomal dominant genetic conditions into routine practice across a range of primary care settings.

## Methods

### Study design

This project builds on previous research from this group involving the development, implementation and evaluation of a primary-tertiary shared care model to improve detection of FH through cascade genetic testing in primary care. The protocol and process for selecting implementation strategies have been described elsewhere.^19 20^ Briefly, the development and implementation of the model followed a 4-phase process according to the Exploration, Preparation, Implementation, Sustainment (EPIS) framework.^21^ The EPIS framework guides projects through key stages of the implementation process and focuses on factors within and across levels (eg, system, organisational, individual levels) that might influence implementation success. During Phase 1 (ie, exploration), a national primary-tertiary shared care model was developed by this research group to help improve detection rates for FH.^15 19^ Phase 2 (ie, preparation) involved tailoring the shared care model and implementation supports to local needs. During Phase 3 (ie, implementation), the model was implemented and evaluated in a real-world setting, and Phase 4 (ie, sustainment) focused on maintaining and scaling up the shared care model. This paper describes Phase 2, whereby a qualitative approach (ie, semistructured interviews) was used to tailor and refine the model. Learnings provided through tailoring the shared care model and supports were then used to develop a generic shared care model that could be applied to a range of other treatable genetic conditions. This paper follows the 32-item checklist of the consolidated criteria for reporting qualitative research (online supplemental material 1).^22^

### Participants

A purposive sample of key stakeholders that would be involved in implementing the primary-tertiary shared care model was sought to provide a range of perspectives based on: mix of early to late career providers, those working with culturally diverse populations, medical and non-medical health professionals, GPs and hospital-based providers, index cases and relatives that had experienced genetic testing in primary and tertiary care settings. Subsequently, snowball sampling was undertaken by asking initial participants to recommend other potential participants (ie, colleagues) that would be involved in implementing the primary-tertiary shared care model to invite to the study. Eligible participants were contacted by email invitation and included GPs, physicians, cardiologists, genetic counsellors and patient and consumer representatives.

### Semistructured interviews

Semistructured interviews were conducted online via the Microsoft Teams videoconference platform. Interviews were undertaken by a female postdoctoral research fellow (KLB) experienced in qualitative research who had no relationship with participants prior to the interviews. The interviewer’s job title and the purpose of the study were described, and participants provided verbal consent, prior to the start of the interview. Two semistructured interview guides were used: one for the patients’ perspective and one for the providers’ perspective. Interview guides were informed by the EPIS framework and literature on implementing genetic testing in primary care to ensure key areas of the primary-tertiary shared care model were addressed. However, the interviews were also guided by the background and responses of the participant to gain a deep understanding from all stakeholders. Prior to the interview, participants were provided with a flow chart of the model of care.^19^ During the interview, the flow chart was explained, and participants were provided with the opportunity to ask questions and comment. Participants were also asked to comment on how they saw this model working within their practice setting (for healthcare providers) or living situation (for patient and consumer representatives). The interviews ranged from 30 to 60 min in duration and were audio recorded and transcribed verbatim. Participants received a voucher for their time. One participant opted to participate in a follow-up interview to provide additional feedback on the shared care model.

### Data analysis

Interviews were audiorecorded and transcribed using Microsoft Teams. NVivo software was used to undertake a reflexive thematic analysis following Braun and Clarke’s 6-phase process.^23^ This approach was chosen as it allowed the lead researcher to reflect and thoughtfully engage with the data using a defined, yet flexible methodology, to ensure a rich understanding of the data. Stage 1 began with data familiarisation, whereby all audio files were listened to entirely while the lead researcher recorded initial thoughts and ideas. During Stage 2, three researchers (KLB, CH and MS) commenced analysis of data using an open coding process in which transcripts were repeatedly reviewed and initial codes developed in relation to the research question. In Stage 3, initial themes were generated with analysis conducted in an iterative, non-linear manner whereby the lead researcher revisited earlier stages to refine or revise potential themes. An inductive approach was used to generate overarching themes that arose across interviews. As themes and subthemes were generated, a collaborative and reflexive approach was then applied whereby three researchers (KLB, CH and MS) reviewed progress to ensure an in-depth understanding of the data and themes generated. The process of familiarisation, coding of the data and generation of themes helped to tailor the primary-tertiary shared care model and set the foundation for designing the generic model of care. Although Braun and Clarke question the use of thematic saturation when using reflexive thematic analysis, we achieved data saturation at 10 participants.

### Patient and public involvement

Patient and public involvement in the design of this research began before the research grant funding application stage. Key stakeholders from NSW, Australia, were consulted to help codesign the shared care model. Patients with FH were involved in the design of this study from the initial planning stages and provided their input through interviews and participation on the Project Steering Committee.

## Results

10 of 14 invited stakeholders participated in an interview (71%). Interviews were conducted over a 5-month period (June–October 2023). Participant demographics are included in table 1.

**Table 1:** Participant demographics

Participant		n (%)
Sex	Male	3 (30)
	Female	7 (70)
Profession	General practitioner*	4 (40)
	Allied health	3 (30)
	Patient	2 (20)
	Other medical specialist	1 (10)

* One participant was interviewed from the perspective as both patient and provider.

Three main themes with 11 subthemes were identified (table 2) that reflect the stages of moving from a usual care to a tailored primary-tertiary shared model of care. Extracts of the data (ie, quotes from participants) have been used to illustrate the described themes and subthemes.

**Table 2:** Themes and subthemes

Theme	Subtheme
Current process for diagnosis and management	Diagnostic pathway Management post genetic testing
Challenges with FH genetic testing in primary care	Knowledge of and awareness of FH and genetic testing Primary care dynamics Working through the hospital system Access to genetic counsellors
Components needed to enable a tertiary-led shared model of care	Streamlined and clear two-way communication pathways Build knowledge and awareness of FH Mechanisms to support further cascade testing Strategies for adding patients to the FH registry Empowering patients and healthcare providers

FH, familial hypercholesterolaemia.

### Theme 1: current process for diagnosis and management

Participants described the current process for the diagnosis and management of FH, beginning with a suspected FH case to ongoing care.

#### Subtheme (1): diagnostic pathway

Participants described the current diagnostic pathway, with suspected cases with FH commonly referred to a genetic or lipid specialist for genetic testing. These referrals were received from primary care or other medical specialists.

Individuals identified to have high LDL cholesterol levels …and they are referred to our service by a GP or by another specialist for review either of their treatment or if there’s an interest in doing some genetic testing to see whether there’s a genetic diagnosis of FH. (Participant 2)

So, if I find someone, I think is an index case, I would send them, in my case, to the RPA [hospital] clinic for them to decide if genetic testing is done. That’s my understanding is that’s the way we’re supposed to do it. (Participant 7)

The diagnostic pathway also included notification of at-risk relatives (ie, either directly or indirectly) and included verbal notification (ie, patients were told to make relatives aware of their risk) or written notification (ie, in the form of a family letter). However, participants in both primary and tertiary care described a low level of cascade genetic testing beyond the first patient (ie, generation of new cases via genetic testing of relatives). In primary care, this was often related to limited knowledge of the MBS Item or that it only recently became available.

we haven’t done very much genetic testing because it’s only very recently become accessible in our setting. (Participant 3)

In tertiary care settings, despite recommendations to notify at-risk relatives via direct or indirect contact, the number of genetic tests generated was less than anticipated.

I would say not as much as I would like [re. cascade genetic testing] though I think I spend a lot of time talking to index cases, mostly about their diagnosis and I strongly encourage them to share information with their relatives. And I sometimes send information directly to relatives. If the proband asked me to. So, yeah, but not as much as I’d like. I would like to say, I guess by the numbers, I would hope that I would be seeing more people for cascade testing than for proband testing. But it’s definitely not the case. (Participant 1)

#### Subtheme (2): management post genetic testing

While participants indicated tertiary care specialists were responsible for diagnosis of FH, management of patients was often described as being the responsibility of GPs.

Once they’re diagnosed by a cardiologist, often the GP will be doing the ongoing management of the patient and the monitoring of their cholesterol. So, it often becomes the GPs role to actually monitor patients. (Participant 10)

Some participants described initial management commencing in tertiary care by multidisciplinary teams with ‘handover’ to GPs for long-term management.

We are very fortunate our clinic [regarding management] has got many health professionals. We have a genetic counsellor, [lipid, endocrinology] specialists…. we also have dietitians and a research nurse. (Participant 1)

We need to plan exit strategies, otherwise the clinics will be just chock-a-block. So, what we do is we follow them up intensely in the first few months…I usually call the GP and provide a verbal handover. (Participant 4)

### Theme 2: challenges with genetic testing for FH in primary care

Participants described several challenges across the stages of the shared care model (ie, from initial patient contact to return of genetic test results) that could hinder successful implementation.

#### Subtheme (1): knowledge and awareness of FH and genetic testing

For several participants who work in primary care, a general lack of awareness of FH and genetic testing was described. This is highlighted in comments related to a limited understanding of FH or MBS Items for genetic testing.

No, because my understanding is that the genetic testing isn’t available or funded through general practice*.* (Participant 7)

#### Subtheme (2): primary care dynamics

Further challenges described by participants related to the dynamic nature of primary care including patients visiting different GPs or lacking a regular GP, family complexities, varying levels of health literacy and GPs having limited time.

I am not being as thorough because I’ve got four patients waiting in the waiting room or, you know, all of that has to be part of it, because you know, when you’re on the clock in general practice, there’s not much time, you’ve got 15 min for everybody*.* (Participant 8)

#### Subtheme (3): working through the hospital system

Participants further highlighted challenges with the hospital system including turnaround time for genetic test results (ie, private vs public pathology services) and clarity or confusion on care provided (or not provided) by tertiary healthcare providers.

My dilemma, so far, is that my experience, just send your patients to the clinic with quite high Dutch lipid scores. None of them have even had a genetic test done, so they’ve come back to me saying manage their risk factors and I go, yes, I thought I was trying to do (that) anyway. I’m thinking yes, but they haven’t (been genetically tested) so I’m not really sure why*.* (Participant 7)

#### Subtheme (4): access to genetic counsellors

Finally, a limited access to genetic counsellors was highlighted as a key barrier to the success of the model.

GPs don’t have access to genetic counsellors, and I checked with Medicare 2 weeks ago, Medicare item number is not available to genetic counsellors. So that is a major barrier for GPs, how do we provide the cascade screening if the GPs do not have access to the genetic counsellors*.* (Participant 3)

### Theme 3: components needed to enable a tertiary-led shared model of care

While discussing factors that might hinder the model, several participants reflected on strategies to address identified challenges that are important to support successful implementation and sustainment of the shared care model.

#### Subtheme (1): streamlined and clear two-way communication pathways

A frequent comment mentioned by participants was the importance of simple and clear guidelines to enable FH genetic testing and management in primary care. Participants from primary care described uncertainty around the referral process, highlighting that it should be easy for GPs to follow and include step-by-step guidelines for ordering genetic tests and providing genetic counselling.

it’s got to be simple, and, you know, I think GPs are pretty overwhelmed with the amount of information because everyone thinks that their condition is the most important condition and so everyone is providing huge amounts of resources and we get a bit resource overloaded. So, I think resources are good, but they’ve just got to be in a place that’s easy to access*.* (Participant 10)

Clear communication between primary care and tertiary care was emphasised as very important. Participants suggested a direct GP line to the clinic would be a helpful resource for additional support.

If I’m stuck, I need a phone number to ring to like smooth it over. (Participant 7)

The streamlined and clear communication also encompassed an element of relationship building as suggested when genetic counsellors contacted GPs directly (or vice versa) to discuss a patient and the importance of specifically addressing the doctor in correspondence rather than a generic ‘dear doctor’ letter.

I think trying to make it (the letter to GP in cascade testing package) so that the person who it is being sent to feels like it’s being sent to them rather than it being too generic, and, you know, it’s sort of like talking about them as being the person who’s working with the patient. So, it’s more about that relationship*.* (Participant 3)

#### Subtheme (2): build knowledge and awareness of FH

GPs were open and discussed opportunities to build knowledge and awareness of FH through educational opportunities including fact sheets (for patient and provider), online resources (eg, HealthPathways, an online health information portal for local GPs) and continuing education opportunities.

I think that’s where things like HealthPathways can be useful, though, because you can link to models and resources through HealthPathways and that can be quite a useful way of getting that information there and making the model work. (Participant 10)

#### Subtheme (3): mechanisms to support further cascade testing

Evidence-based guidelines for FH care include offering cascade testing to at-risk relatives. For that reason, participants were asked how the model of care would support ongoing cascade testing through primary care. Participants described several approaches including primary care notification of relatives (both direct and indirect), providing guidance to patients and clinicians to conduct further cascade testing of relatives and incentivising GPs to do cascade testing.

I guess you could still get consent from that original patient, like you could ask for consent to contact family members or like disclose that if you see the family members for another reason. So that could be another way you could do it*.* (Participant 6)

I think it would just be explaining to them, who needs to get tested and asking them to contact those relatives. But I mean, you know, if there was some sort of handout that you could give to patients saying, you know, these are the relatives you should contact and you should tell them, I think the piece of information that is most useful is who to contact*.* (Participant 6)

#### Subtheme (4): strategies for adding patients to the National FH Registry

An integral component of the primary-tertiary shared care model to enhance the care of individuals and families with FH includes maintaining a National FH Registry. The registry is intended to support ongoing FH research to advance understanding of FH, improve treatment options and enhance patient care. Feedback suggested an invitation to the registry could be done at different stages such as part of the cascade testing process or post results conversation.

Also building it into all of this somehow as a tick box thing to get that consent at an early phase, because even when I signed the forms for the genetic counsellor, you know if she said. Oh, can you, if you are positive, would you be part of the database and I’d happily just sign another form at that starting point. (Participant 8)

#### Subtheme (5): empowering patients and healthcare providers

The final theme described in interviews was the importance of providing support by empowering both patients and providers to improve the success of a shared care model. This was highlighted by one participant who described the central role of the patient in a shared care model.

when you have a constant, every one of those interactions has a patient in it, and it’s the same patient, or patient family unit. We could probably play, if we’re empowered to, could play a much stronger role in the care coordination. (Participant 5)

While several GPs described their ability to handle various healthcare issues, including genetic testing, they still required guidance and support to feel confident in performing some of these tasks.

what makes the job interesting in general practice is being able to do as much as you can, you know, like I’ve treated Hep C and I love that because I don’t need to refer to treat Hep C, I can treat Hep C because I’m empowered to do that through really good guidelines and really good guidance. (Participant 8)

### Tailoring of the shared care model

Feedback from interviews supported tailoring and refining the primary-tertiary shared care model to meet the local contextual needs of healthcare providers and patients. Using feedback from key stakeholders, two support packages were developed and include: (1) a cascade testing package and (2) a post results package. During interviews, the two packages were presented with participants providing further feedback regarding content and any suggested changes or additions. The final cascade testing package includes the following: (1) a one-page cascade screening guide for the GP, (2) a prefilled pathology request form, (3) a genetic testing consent form, (4) a patient FH factsheet and (5) links to GP resources and contact details for the clinic for additional support. A post genetic test result package for a positive result includes the following: (1) results letter for GP, (2) results letter for patient, (3) family letter (for the patient to pass on to relatives and facilitate further cascade testing) and (4) a National FH Registry consent form.

### Development of a generic shared care model

**Figure 1:**
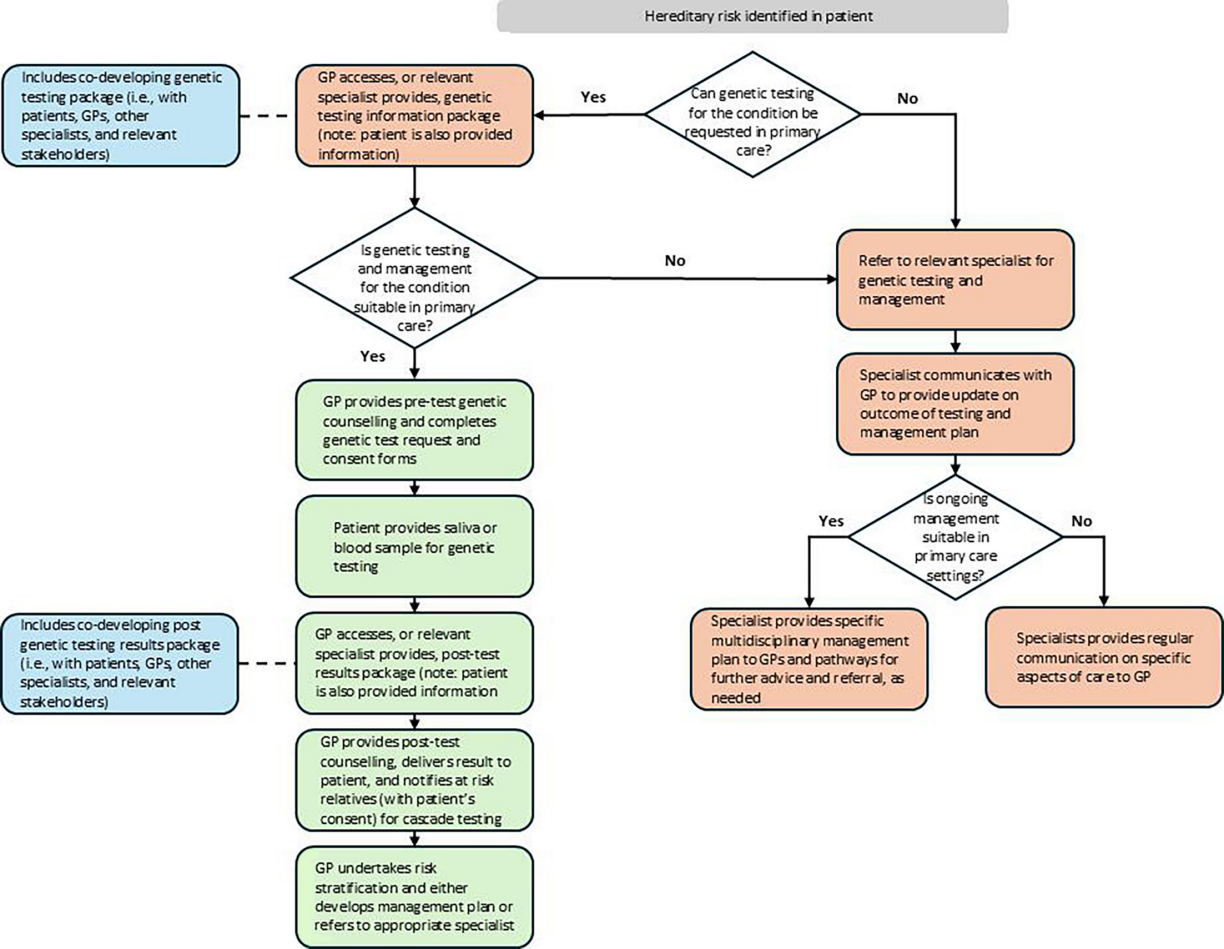
Generic shared care model to be generalised to specific treatable autosomal dominant genetic conditions. GP, general practitioner.

Learnings from developing and tailoring the primary-tertiary shared care model for FH were used to design a generic shared care model that can be generalised to specific treatable autosomal dominant genetic conditions. It is important to emphasise the importance of a co-designed approach that includes end users (ie, GPs and other medical specialists and patients) when adapting the generic model to specific genetic conditions. For example, support packages should be designed, piloted and refined through feedback from stakeholders to develop key elements of the shared care model necessary for specific genetic conditions. The generic shared care model can be seen in figure 1.

## Discussion

To keep pace with the rapid expansion in genomic medicine, healthcare systems need to be prepared to handle the growing demand for genetic services. A shortage of genetic professionals internationally highlights that genetic specialists will not have capacity to handle the diagnosis and management of all genetic conditions.^24^ Solutions to address these challenges are needed to ensure all populations can access timely, equitable and affordable genetic services. Using implementation science, this study describes a process for integrating genetic testing into primary care and thereby offers an alternate service delivery model by building capacity within primary care.^25^

Recognising the increasing demand for accessible and affordable genetic services, several models of care have been developed that include integrating genetic counsellors into primary care, telehealth genetic services, multidisciplinary genetic clinics and building capacity for non-genetic specialists to screen, diagnose and manage genetic conditions.^1926^,^32^ These models aim to improve patient care by providing efficient, timely and better access to genetic services and share a common theme that uses a collaborative team approach. However, despite primary care presenting an ideal opportunity to integrate genetic medicine into routine care, it is less commonly done, with most service models led by genetic specialists.^29 33^ The development of our generic shared care model focuses on primary care and is similar to previous research using implementation science methods to improve screening for FH.^26^ Our model was designed using a collaborative approach with input from a range of stakeholders (ie, genetic specialists, lipid specialists, GPs, patients).

Mainstreaming genetic testing, a process of upskilling non-genetic specialists to order genetic testing,^34^ is an important first step to integrating genomic medicine into routine clinical practice. A key component of our model is supporting GPs by providing genetic counselling support as needed, developing resources and working closely with patients and care providers. However, developing skills, confidence and knowledge around genetic testing takes time and, as such, beginning with genetic conditions that are easy to manage in primary care is a logical approach. The model allows GPs to develop and build skills and, with time, will increase confidence in delivering genomic medicine. Our model of care is in line with recommendations to build workforce capacity to support the integration of genetic medicine into routine clinical care.^3 10^ This model of care was developed for FH, a genetic condition that can be treated using routinely prescribed medications independently managed by GPs. The approach is particularly well suited for highly penetrant autosomal dominant conditions given the high return on investment for cascade testing (ie, 50% of first-degree relatives likely to have the condition) and initiating early treatment (ie, high likelihood that patients with an FH gene change will experience high LDL cholesterol). The model focuses on genetic cascade testing, given the newly introduced MBS Item, which can be requested by GPs in Australia. However, our model is also generalisable for phenotypic testing, as we provide information to GPs to support phenotypic testing within our cascade testing support package, if patients do not consent to genetic cascade testing. While it is generalisable to phenotypic testing, the absolute certainty of genetic diagnosis is extremely helpful in this process, which is time and effort-intensive for both clinicians and patients.

Several barriers and enablers to the integration of genomic medicine into routine care have been identified across the provider, organisational and system level, which present multiple challenges to the implementation of evidence-based care.^15^ Implementation science recognises the complexity of healthcare systems and offers a structured and systematic approach to implementing evidence-based guidelines into practice;^35^ however, its application in genomic medicine remains limited.^36^ Several recent papers highlight the importance of including implementation science in the design and conduct of research studies focused on integrating genomic medicine into routine clinical practice.^1837^,^40^ In the context of genetic testing, implementation science approaches have been used to bridge gaps in care associated with limited knowledge and awareness, workforce capacity, limited access to genetic services or fears about insurance coverage.^15 19 26^ Our primary-tertiary shared care model for FH was informed by implementation science methods and incorporated feedback from those involved in implementing the model at all stages of development. A key element of success is the active involvement of end users in the process. A similar implementation science approach is necessary to mainstream genomic medicine for other genetic conditions into routine clinical practice.^3^

The tailoring of our shared care model was guided by end user feedback provided during interviews. Several challenges were described by participants with respect to the shared care model and how it might work within their local settings. In line with previous research, GPs felt they could carry out genetic testing but described limited time, lack of knowledge and awareness of genetic testing and access to genetic expertise as an issue.^7 26 41^ Mechanisms to address these challenges within our shared care model include: (1) direct contact with patients to navigate options for those without a GP, (2) direct contact with the GP by tertiary care clinicians, (3) a direct line to the genetics clinic for both GPs and patients, (4) providing two support packages to help in diagnosis and management, (5) professional development opportunities^42^ and (6) providing resources to address health literacy in plain language or utilising translation services. The development and refinement of our two support packages is an approach that is in line with previous research using implementation science methods to develop a package to support FH screening in primary care.^26^ Components of our support packages are in accordance with previous research where GPs describe resources that would help integrate genomic medicine into practice including contact details for genetic services, genetic referral information and testing criteria, and direct contact with local genetic counsellors for questions.^8^ While the development of the support packages was specific to FH, tailoring of the package to support the model of care can be generalised to other genetic conditions and adapted to local context and different healthcare settings as the challenges identified in our study are likely applicable to other common and treatable autosomal dominant genetic conditions.

### Strengths and limitations

This study has several strengths. First, engaging with key stakeholders for feedback on how to tailor the model to suit their clinical environment is an important part of success as it helps to support end user buy-in. Second, we sought to include stakeholders across the care spectrum with a range of experience or knowledge of FH to ensure the appropriate supports were available regardless of the level of experience. Although our model of care for FH was developed based on Australian conditions, our process model can be used as a guide and modified to fit within local requirements and healthcare settings in other countries. Finally, our study used a systematic approach guided by implementation science methods to develop and tailor a model of care which will enhance the likelihood of success.

There are some potential limitations to this study. The full range of stakeholders who could provide feedback on the model of care may not have been represented. Despite holding discussions with representatives from private sector pathology labs, none participated in an interview. Our generic model of care was developed from a shared care model focused on FH, a genetic condition that is easy to manage in primary care. As such, there may be additional barriers to adapting it to other genetic conditions. However, the use of implementation science methods that engage key stakeholders will help address these challenges and ensure the model can be tailored and implemented to various genetic conditions and across healthcare settings.

Further research is needed to determine whether a similar approach to our model is suitable for other genetic conditions such as BRCA 1/2 that introduce greater clinical complexity and implications for genetic counselling, access to which is limited to GPs in Australian primary care settings. Furthermore, genetic conditions that require multidisciplinary management (eg, polycystic kidney disease) and those with increased psychosocial burden, either for patients or their families, will require further investigation to pave the way for pathways to genetic mainstreaming. Low GP awareness presents an important limitation to implementation, likely varying between urban and rural settings. Monitoring GP ordering patterns for the MBS Item over time could provide insight into this. The issue around restricted access to genetic counsellors applies to all aspects of medical care, not just FH, particularly in rural and remote locations, and might be mitigated by leveraging the increased acceptance and infrastructure of telehealth established post-COVID-19. The relative feasibility of implementing a primary-tertiary care model for FH cascade testing raises the importance of this exemplar condition for achieving genetics mainstreaming in Australia and internationally. Finally, future research should determine the barriers and facilitators to the management of FH after detection and how risk reduction pathways can be implemented for paediatric FH to ensure that the benefits of early detection are realised.

## Conclusion

Our generic model of care demonstrates how GPs and specialists can work together to design, adapt and deliver an effective shared care model for genomic medicine. This paper adds to the increasing call to incorporate genomic medicine into routine primary care and illustrates how a collaborative approach to design and implementation will enhance utilisation and uptake. Our primary-tertiary shared care model provides a process model that can be adapted to a range of genetic conditions and will support and build capacity for GPs to be engaged, empowered and confident in carrying out genomic medicine as part of routine care.

## Supplementary material

10.1136/fmch-2024-003258 online supplemental file 1

## Data Availability

Data are available upon reasonable request.
