# Supplementary material for: Process for mainstreaming genetic cascade testing into primary and tertiary health systems in familial hypercholesterolaemia (FH), an autosomal dominant, fully penetrant disorder
Source: Fam Med Community Health. 2025 Aug 14;13(3):e003258. doi: 10.1136/fmch-2024-003258 (PMC12352198; doi:10.1136/fmch-2024-003258)
Supplement: online supplemental file 1 [file fmch-13-3-s001.pdf]

Supplementary material 1. Consolidated criteria for reporting qualitative studies (COREQ): 32-item checklist

No	Item	Guide questions/description	Found on (manuscript page)
**Domain 1: Research team and reflexivity**			
Personal Characteristics			
1.	Interviewer/facilitator	Which author/s conducted the interview or focus group?	6
2.	Credentials	What were the researcher's credentials? *E.g. PhD, MD*	6
3.	Occupation	What was their occupation at the time of the study?	6
4.	Gender	Was the researcher male or female?	6
5.	Experience and training	What experience or training did the researcher have?	6
Relationship with participants			
6.	Relationship established	Was a relationship established prior to study commencement?	6
7.	Participant knowledge of the interviewer	What did the participants know about the researcher? e.*g. personal goals, reasons for doing the research*	6
8.	Interviewer characteristics	What characteristics were reported about the interviewer/facilitator? e.g. *Bias, assumptions, reasons and interests in the research topic*	6
**Domain 2: study design**			
Theoretical framework			
9.	Methodological orientation and Theory	What methodological orientation was stated to underpin the study? *e.g. grounded theory, discourse analysis, ethnography, phenomenology, content analysis*	7
Participant selection			
10.	Sampling	How were participants selected? *e.g. purposive, convenience, consecutive, snowball*	6
11.	Method of approach	How were participants approached? e.*g. face-to-face, telephone, mail, email*	6
12.	Sample size	How many participants were in the study?	8
13.	Non-participation	How many people refused to participate or dropped out? Reasons?	8
Setting			
14.	Setting of data collection	Where was the data collected? e.*g. home, clinic, workplace*	7
15.	Presence of non-participants	Was anyone else present besides the participants and researchers?	No
16.	Description of sample	What are the important characteristics of the sample? *e.g. demographic data, date*	8
Data collection			
17.	Interview guide	Were questions, prompts, guides provided by the authors? Was it pilot tested?	6
18.	Repeat interviews	Were repeat interviews carried out? If yes, how many?	7

19.	Audio/visual recording	Did the research use audio or visual recording to collect the data?	7
20.	Field notes	Were field notes made during and/or after the interview or focus group?	7
21.	Duration	What was the duration of the interviews or focus group?	7
22.	Data saturation	Was data saturation discussed?	Yes
23.	Transcripts returned	Were transcripts returned to participants for comment and/or correction?	No
**Domain 3: analysis and findings**			
Data analysis			
24.	Number of data coders	How many data coders coded the data?	3
25.	Description of the coding tree	Did authors provide a description of the coding tree?	No
26.	Derivation of themes	Were themes identified in advance or derived from the data?	7-8
27.	Software	What software, if applicable, was used to manage the data?	7
28.	Participant checking	Did participants provide feedback on the findings?	No
Reporting			
29.	Quotations presented	Were participant quotations presented to illustrate the themes / findings? Was each quotation identified? e.*g. participant number*	Yes
30.	Data and findings consistent	Was there consistency between the data presented and the findings?	Yes
31.	Clarity of major themes	Were major themes clearly presented in the findings?	Yes
32.	Clarity of minor themes	Is there a description of diverse cases or discussion of minor themes?	Yes
